# Paediatric vision screening by non-healthcare volunteers: evidence based practices

**DOI:** 10.1186/s12909-019-1498-x

**Published:** 2019-02-28

**Authors:** K. Sabri, B. Easterbrook, N. Khosla, C. Davis, F. Farrokhyar

**Affiliations:** 1 Division of Ophthalmology, Department of Surgery, McMaster University, 1200 Main Street West, 3V2, Hamilton, ON L8N 3Z5 Canada; 2 McMaster Pediatric Surgery Research Collaborative, Department of Surgery, McMaster University, 1200 Main Street West, Hamilton, ON L8N 3Z5 Canada; 3 McMaster Paediatric Eye Research Group, Department of Surgery, McMaster University, 1200 Main Street West, 3V2, Hamilton, ON L8N 3Z5 Canada; 4 Hamilton-Wentworth Catholic District School Board, 90 Mulberry Street, P.O. Box 2012, Hamilton, ON L8N 3R9 Canada; 5 Office of Surgical Research Services, Department of Surgery, McMaster University, 39 Charlton Avenue East, Hamilton, ON L8N 1Y3 Canada

**Keywords:** Testing/assessment, Quantitative research Methods, Ophthalmology, Paediatrics

## Abstract

**Background:**

The purpose of this study was to test the sensitivity and specificity of eight undergraduate volunteer examiners conducting vision screening tests in a community setting, in order to determine if non-eye care professionals were able to be trained to an appropriate level of skill.

**Methods:**

Eight undergraduate volunteer examiners were trained to conduct vision screening tests to address a gap in pediatric community eye care. Phase I of the study was implemented in the pediatric ophthalmology clinic, and phase II was conducted in nine local schools. Phase I consisted of 40 h of training for each volunteer regarding specific vision tests. Phase II consisted of screening children at nine local schools.

**Results:**

A total of 690 children from nine local schools were screened by both the volunteer examiners and the optometrist during the course of this study. Volunteer examiners had a screening sensitivity of 0.80 (95%CI 0.66–0.90) and screening specificity of 0.75 (95%CI 0.71–0.78) when compared to the study optometrist. The overall accuracy of volunteer examiners was 75%. The resulting positive likelihood ratio was 3.24 (95%CI 2.6–3.9), indicating that a child with vision impairment was 3.2 times more likely to fail the vision test performed by the volunteer examiners compared to a child with no vision impairment.

**Conclusions:**

Non-healthcare professionals can be trained to an acceptable degree of accuracy to perform vision screening tests on children, which may assist in mitigating existing gaps in paediatric eye care.

## Background

Adequate childhood vision is key for physical, emotional, and social progress throughout the lifespan [1]. Population-based studies have shown the prevalence of common causes of visual impairment among non-infant children in developed countries such as Britain and Australia to be as high as 22.6% for myopia and 11% for astigmatism [2–6]. Similar studies of the prevalence of hyperopia in have found incidence as high as 12.8% in children 5–15 years of age [7, 8]. Undetected and untreated paediatric ocular disorders can lead to reversible and irreversible vision loss later in life, including amblyopia. Amblyopia, which can be cured if treated in early childhood, is the main cause of monocular blindness in the 20–70 year age range in developed countries [9]. Amblyopes have more than twice the risk of non-amblyopes for losing vision in their fellow eye from accident or trauma, becoming visually impaired in both eyes for life [10]. According to the National Coalition for Vision Health, individuals with vision loss have double the risk of mortality, difficulties with daily living and triple the risk of depression [11]. Overall prevalence of low vision and blindness has increased by + 7.3% in children aged 6–17 years in certain countries [12].

Vision screening recommendations begin in infancy and continue every 12 to 24 months throughout childhood [13]. However, in developed nations including Canada, the ability to meet these recommendations depends upon numerous factors including clinician availability, practice patterns, medical training, and community as well as parental acceptance. Without national screening programs, the ability to meet such recommendations remains variable. Despite the importance of identifying children with vision disorders in order to ensure proper and timely treatment, recent reports have shown inconsistent screening across Canada and the United States [14, 15]. While there are community-based screening programs run by organizations such as the National Association of School Nurses (NASN) [16], and the Lions Club International [17], nine states in the U.S.A. and six provinces in Canada have no mandated school-aged vision screening program [15, 18]. To mitigate this substantial gap in paediatric eye care, the aim of this study was to prospectively assess the feasibility, accuracy, sensitivity and specificity of training undergraduate university students to perform specific visual function screening tests in order to detect certain visual disorders in school aged children.

## Methods

### Study design

**Fig. 1:**
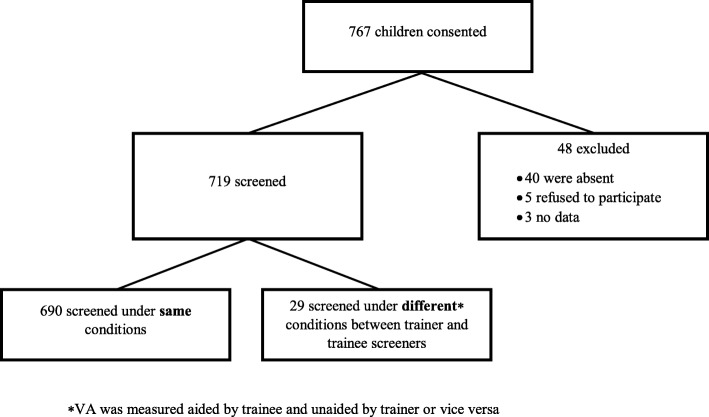
Study Flow Diagram

This was a prospective trial conducted in two phases as part of a school-based vision screening program from March–May 2015. The study population included children aged 4 to 14 years who were enrolled in full-time education at nine Hamilton-Wentworth Catholic District School Board (HWCDSB) elementary schools. Following parental consent, each child received both a full eye examination by the study optometrist and vision screening by one of the eight volunteer examiners (See Fig. 1). All children received a full eye examination by the study optometrist which included a dilated fundus examination and refraction. Full eye exams were provided by the optometrist, rather than screening, as an added benefit to study participation to increase consent rate. Research ethics approval was obtained from both the Hamilton Integrated Research Ethics Board and the Research Advisory Committee of the HWCDSB. All research conducted adhered to the tenets of the Declaration of Helsinki.

### Phase I: Training the vision screeners

Eight undergraduate bachelor’s degree students (four health sciences students, two nursing students, and one each from arts and life sciences) underwent 40 h of training to correctly perform the following tests: distance visual acuity (VA) using Snellen crowded letters or LEA symbols (depending on child’s age); near VA using Rosenbaum chart or LEA symbols; Ishihara color vision; and Randot stereoacuity. All training was conducted by the study optometrist. Volunteer examiners were educated, trained and assessed on the principles and practices of performing vision screening tests, using videos, written instructions, practical exercises and supervised eye examinations, all of which took place at the paediatric eye clinic at McMaster Children’s Hospital in Hamilton, Ontario, Canada.

### Phase II: In-school screening

Research was conducted at nine local Catholic schools in Hamilton (Ontario, Canada) over the course of 8 weeks, including children between 4 and 14 years of age. Schools were randomly selected to be approached for inclusion, and principals had to agree to participate prior to on-site visits. Each child received a full eye exam by the optometrist and vision screening performed by volunteer screeners, in randomly selected sequence. If the optometrist was randomly designated to examine the child first, they performed all tests exclusive of dilated fundus examination and refraction, then completed that portion of the exam after the child had been screened by the volunteers. The M&S Smart System (M&S Technologies, Niles, IL), a computer-based visual acuity testing system where the Snellen chart is shown on a computer screen, was used for measuring VA. Visual Acuity was recorded as the lowest line on which the child correctly identified half or more of the optotypes while the fellow eye was occluded with an adhesive Ortopad orthoptic eye patch (Ortopad USA, Tuscon, AZ). Each child’s distance visual acuity was measured at a distance of 20 ft with Snellen crowded letters, or LEA symbols if the child could not read. Monocular near visual acuity was measured using the Rosenbaum chart at 36 cm or LEA Symbol chart at 40 cm. It was ensured that for any given child, the same test was used by both the optometrist and volunteer for distance visual acuity (Snellen or LEA) and near visual acuity (Rosenbaum or LEA) measurement. Ishihara plates were used for monocular color vision testing and the Randot stereotest was administered for near stereoacuity. Colour vision was scored separately for each eye as the number of plates the child identified correctly. Assessment of colour vision was included in the tests conducted in this study as upwards of 8% of the global male population experiences colour deficiency. Defects in colour vision can often go undetected and may affect a child’s learning [19]. Assessing children’s stereopsis was included in the study as an indirect way of detecting children with manifest strabismus. In cases of manifest strabismus, even if the visual acuity is good in both eyes, the stereopsis will be reduced. Therefore, by testing stereopsis, the volunteer examiners would be able to detect children with suspected strabismus. Stereoacuity tests are part of the screen and refer processes in some vision screening programs in both the US and Canada [20, 21]. If the child wore glasses, all vision screening assessments were performed with the child wearing his or her glasses. None of the children in this study wore contact lenses.

### Data collection and data standardization

Study data were entered into a password protected, encrypted computer database. Data collected included gender, age, school, near visual acuity, distance visual acuity, stereoacuity and color vision.

### Sample size calculation and data analysis

A previously published rate of impaired vision in Hamilton area elementary schools was used to calculate sample size. Assuming that approximately 20% of children enrolled will have vision impairment and an alpha error of 0.05, we needed 353 participants to achieve a confidence interval of + 5% around the expected sensitivity and specificity of 90%.

We decided on an a priori moderate to high level of agreement as acceptable for all phases of the study (ICC of 0.61 or greater) [22]. Intraclass correlation coefficient (ICC) was calculated using two-ways mixed effects models for overall reliability of the volunteer examiners to conduct vision tests compared to the optometrist. ICCs as a measure of reliability with the corresponding 95% confidence intervals (CI) are reported. Sensitivity, specificity, and likelihood ratios with corresponding 95% CIs were reported to measure accuracy of volunteer examiners’ vision screening compared to the study optometrist. SPSS (www.ibm.com) was used for data analysis.

## Results

Overall, 3500 children at nine Catholic elementary schools were approached for participation in this study, of which 767 gave consent. Of the 767 children who initially consented, 48 participants were excluded for the following reasons: 40 were absent from school, 5 refused to participate, and 3 had no data captured. This led to 719 children being screened, of which, 29 participants were further excluded for being screened by optometrist with glasses and by volunteer screener without, or vice versa. Therefore, 690 children participated in the study and were included in the analysis (see Fig. 1). Median age of study children was 8 years old, with a minimum and maximum age of 4 and 14 years, respectively (see Table 1 for demographics).

**Table 1:** Demographic variables

Participant Demographics	Counts (%)
Female	382 (55)
Male	308 (45)
Median age in years (minimum, maximum)	8 (4, 14)
Mean age in years	8.1
Total school children screened	690 (100)

### Agreement between volunteer examiners and optometrist

Table 2 showcases the level of agreement between the volunteer examiners and study optometrist across all vision screening tests conducted. The overall level of agreement for all vision screening tests was greater than the predefined criteria of 0.61 as moderate to substantial agreement. Stereoacuity measurements presented the lowest level of agreement between volunteers and optometrist, with an ICC of 0.65 (95% CI 0.59–0.69). Conversely, the highest agreement coefficient between volunteers and optometrist was seen in colour vision assessment with a result of 0.86 (95% CI 0.82–0.87).

**Table 2:** Level of agreement between trainees and trainer for different vision screening tests

Trainees	Distance VA (Right eye)	Distance VA (Left eye)	Near VA (Right eye)	Near VA (Left eye)	Color (Right eye)	Color (Left eye)	Stereo-Acuity
1	0.91	0.95	0.86	0.94	0.86	0.95	0.89
2	0.78	0.82	0.05	0.55	0.80	0.80	0.10
3	0.75	0.85	0.86	0.91	0.40	0.39	0.76
4	0.73	0.52	0.64	0.64	0.86	0.86	0.69
5	0.71	0.92	0.89	0.78	0.98	0.95	0.89
6	0.61	0.60	0.64	0.71	0.67	0.59	0.14
7	0.44	0.61	0.59	0.58	0.80	0.72	0.47
8	0.24	0.40	0.61	0.60	0.79	0.84	0.32
Total	0.68	0.79	0.65	0.74	0.85	0.86	0.65

### Accuracy and limitations of volunteer examiners as vision impairment screeners

Commonly accepted guidelines for vision screening failure criteria are a presenting distance visual acuity of 20/30 or worse in one or both eyes for children > 5 years of age, a presenting distance visual acuity of 20/40 or worse in one or both eyes in children < 5 years of age, or a difference of two or more lines in presenting distance VA between the two eyes [13, 20]. Using these guidelines, 6.6% (4.9–8.8%) of 690 children failed vision test by the optometrist. Table 3 shows the data for the volunteer examiners’ diagnostic ability to correctly screen children who have vision impairment.

**Table 3:** Accuracy of Trainee Screeners^a^

	Children failed by trainer	Children passed by trainer	Total
Children failed by trainees	37	160	197
Children passed by trainees	9	484	493
Total	46	644	690

^a^Based on failure criteria of best corrected distance VA < 20/30 if the child was > 5 years old, best corrected distance VA of < 20/40 if the child was < 5 years old, or > 2 lines difference between eyes

Prevalence: 6.6% (4.9–8.8%)

Accuracy: 75% (71–78%)

Sensitivity: 0.80 (0.66–0.90)

Specificity: 0.75 (0.71–0.78)

Likelihood Ratio of a positive test: (sensitivity/[1-specificity]) = 3.24 (2.66–3.94)

Likelihood Ratio of a negative test: ([1-sensitivity]/specificity) = 0.26 (0.14–0.47)

Volunteer examiners had a high screening sensitivity of 0.80 (95% CI 0.66–0.90) and a screening specificity of 0.75 (95% CI 0.71–0.78). This resulted in a positive likelihood ratio of 3.24 (95% CI 2.6–3.9), indicating that a child with vision impairment was 3.2 times more likely to fail the vision screening performed by the volunteer examiners when compared to a child without vision impairment. Overall volunteer examiner accuracy in correctly identifying eyes with and without vision impairment was 75% (71–78%).

### Economic and other implications

This study provides further evidence of the potential cost benefits associated with utilization of non-healthcare professional vision screeners when compared to use of health care professionals such as ophthalmologists, orthoptists, optometrists, paediatricians and nurses. For this study, the total cost of conducting the school vision screening was $23,900.00 CAD. The cost was broken down as such: $10,000 (2 x $5000 for M&S systems) + $12,000 for trainer optometrist salary during training and eye examinations + $1200 for Ortopad patches + $400 for Ishihara color plates ($200 each) + $300 for Randot stereoacuity booklets ($150 each) + $0 for volunteer examiners. This equates to an approximate cost of $35 per child screened. Almost 50% of the cost was equipment such as M + S systems, which were one time, start-up costs. After removing start-up and equipment costs, subsequent screening of the same number of children would cost approximately $19 per patient. During this study, there was no extra cost associated with volunteer examiners being able to screen at the schools. In future, clinical insurance for volunteers or covering the cost of vulnerable sector screening could be included as cost considerations.

## Discussion

This large, prospective study provides evidence of the ability to train non-healthcare professionals to a high standard of accuracy in performing specific paediatric vision screening tests when compared to an optometrist, while requiring minimal economic investment. The results are promising encouragement that undergraduates can be trained to an acceptable degree of sensitivity to perform specific vision screening tasks. This also provides detailed evidence regarding which vision screening tests volunteer examiners perform well, and which tests may require more extensive training prior to future school-based screenings. While most vision screening programs do not include testing of colour vision, some have begun using stereopsis in their vision screening recommendations, as these tests can add useful information regarding visual function [20, 21]. Therefore, in this study, we did assess the accuracy of volunteer screeners in performing such tests, as they may have a place in future vision screening programs. The variations in performance between individual volunteer examiners in conducting the vision screening tests may be due to a variety of factors including differing levels of baseline knowledge, varying aptitude in learning practical skills and differing levels of comfort communicating and dealing with young children.

It is important for further research to be conducted regarding the efficacy of non-eye care professionals and non-healthcare professionals administering vision screening tests, as well as the parental acceptability of volunteer screeners. Healthcare professional alternatives for vision screening have included general practitioners, paediatricians [23–25], medical students and nurses [26, 27], while non-healthcare professional alternatives have included self-screening, undergraduate students or school teachers who were taught how to appropriately screen for ocular diseases [28–31]. While it has been recommended that primary care physicians and paediatricians provide initial vision screening examinations [25], it is not feasible to assume that these healthcare professionals have the available time within current healthcare contexts to provide such extensive services. This is one reason why community-based programs rely on volunteer laypersons to conduct screening [31]. Recent technological advancements have expanded the possibility of a larger breadth of vision tests to be conducted by non-healthcare professionals for both adults and children alike. This may simultaneously decrease the cost of screening, increase accessibility and the accuracy of vision screening by allowing mobile screeners to assess a larger proportion of the population who may be otherwise denied access to eye care.

New vision screening devices such as the Paediatric Vision Screener [32] and the Spot Vision Screener [33] are automated screening tools with a one-time cost associated with purchase. Once the device is purchased, concomitant costs related to training and vision screenings are minimal. These electronic devices can be programmed to detect specific risk factors for different conditions, or to simply differentiate which children should be referred to an optometrist for further assessment. The continuous improvements being made in technology, when coupled with the decreasing costs of hardware, mean that automated vision screening devices are becoming increasingly affordable and can be used by non-healthcare volunteer vision screeners. In this way appropriately trained non-health care professionals equipped with the necessary vision screening devices, can provide a very favourable method of vision screening for children which is scalable, affordable and accessible for all communities. Future research includes the development of a predictive model to determine the duration and intensity of training required for each prospective volunteer examiner based on their individual characteristics and aptitudes. Another area of future research includes assessing the sensitivity and specificity of undergraduate students utilizing modern technology such as Paediatric Vision Screener and Spot Vision Screener tools to better assess vision disorders in paediatric patients.

### Limitations and other considerations

The primary limitation of this study was the fact that all volunteer examiners received the same level of training in performing the vision screening tests, irrespective of possible individual variations in their competence and uptake of clinical skills. A secondary limitation of this study was a consent rate of 22%. While this is not a high rate of consent, we were unable to explore why so many parents declined to consent for this study. One potential reason could be a lack of confidence regarding the abilities of volunteer vision screeners.

Additionally, it is important to note that the visual function tests included in the screening process will not detect all possible causes of impaired vision such as moderate or high hyperopia. When measuring colour vision, the small differences in trainer/trainee agreement between left and right eyes likely comes down to the interest and energy level of the individual children engaging in these tests. In future it may be better to test colour vision binocularly. Finally, future studies could focus on increasing efficiency of training: reduce training period to less than 40 h while maintaining or improving trainee sensitivity and specificity.

## Conclusion

The results of this study suggest that undergraduate non-healthcare professionals can be trained to an acceptable level of accuracy when conducting specific vision screening tests on school children. This study provides further evidence-based directions for paediatric vision screening by non-healthcare professionals. It is hoped that with further research, paediatric vision screening by non-healthcare professionals can be developed into an increasingly evidence based, large scale, viable, accurate and cost-effective method of screening that can be taught and implemented in a timely manner. This would significantly reduce costs and increase accessibility of eye care for children worldwide; not only in school settings, but rural and underdeveloped areas as well.
